# Effect of the Phosphodiesterase 5 Inhibitor Sildenafil on Ischemia-Reperfusion-Induced Muscle Mitochondrial Dysfunction and Oxidative Stress

**DOI:** 10.3390/antiox8040093

**Published:** 2019-04-07

**Authors:** Liliane Tetsi, Anne-Laure Charles, Isabelle Georg, Fabienne Goupilleau, Anne Lejay, Samy Talha, Myriam Maumy-Bertrand, Claire Lugnier, Bernard Geny

**Affiliations:** 1 Unistra, Fédération de Médecine Translationnelle, Equipe d’Accueil 3072, « Mitochondrie, Stress oxydant et Protection Musculaire », Institut de Physiologie, 67000 CEDEX, France; gemmelere@yahoo.fr (L.T.); anne.laure.charles@unistra.fr (A.-L.C.); isabelle.georg@unistra.fr (I.G.); goupilleau@unistra.fr (F.G.); anne.lejay@chru-strasbourg.fr (A.L.); samy.talha@chru-strasbourg.fr (S.T.); claire.lugnier@unistra.fr (C.L.); 2 Hôpitaux Universitaires de Strasbourg, Service de Physiologie et d’Explorations Fonctionnelles, 67000 Strasbourg, France; 3 Hôpitaux Universitaires de Strasbourg, Service de Chirurgie vasculaire et de transplantation rénale, 67000 Strasbourg, France; 4 IRMA, équipe MoCo et LabEx IRMIA, 7 rue René Descartes, 67084 Strasbourg CEDEX, France; mmaumy@math.unistra.fr

**Keywords:** cyclic nucleotide phosphodiesterase, sildenafil, muscle, ischemia, reperfusion, peripheral arterial disease, mitochondria, reactive oxygen species, oxidative stress, calcic retention capacity

## Abstract

Lower-limb ischemia-reperfusion (IR) is frequent and associated with significant morbidity and mortality. Phosphodiesterase 5 inhibitors demonstrated antioxidant and beneficial effects in several organs submitted to IR, but their effects on muscle mitochondrial functions after lower-limb IR are unknown. Unilateral hindlimb IR (2 h tourniquet followed by 2 h reperfusion) without or with sildenafil (1mg/kg ip 30 minutes before ischemia) was performed in 18 mice. Maximal oxidative capacity (V_Max_), relative contribution of the mitochondrial respiratory chain complexes, calcium retention capacity (CRC)—a marker of apoptosis—and reactive oxygen species (ROS) production were determined using high-resolution respirometry, spectrofluorometry, and electron paramagnetic resonance in gastrocnemius muscles from both hindlimbs. IR significantly reduced mitochondrial V_Max_ (from 11.79 ± 1.74 to 4.65 ± 1.11 pmol/s*mg wet weight (ww), *p* < 0.05, −50.2 ± 16.3%) and CRC (from 2.33 ± 0.41 to 0.84 ± 0.18 µmol/mg dry weight (dw), *p* < 0.05; −61.1 ± 6.8%). ROS tended to increase in the ischemic limb (+64.3 ± 31.9%, *p* = 0.08). Although tending to reduce IR-related ROS production (−42.4%), sildenafil failed to reduce muscle mitochondrial dysfunctions (−63.3 ± 9.2%, *p* < 0.001 and −55.2 ± 7.6% *p* < 0.01 for V_Max,_ and CRC, respectively). In conclusion, lower limb IR impaired skeletal muscle mitochondrial function, but, despite tending to reduce ROS production, pharmacological preconditioning with sildenafil did not show protective effects.

## 1. Introduction

Peripheral arterial disease (PAD) is frequent and associated with significant morbidity and mortality [1,2]. PAD overall prevalence ranges from 3% to 10%, increasing to 15–20% in persons older than 70 years of age [3]. The annual incidence of critical limb ischemia in patients admitted to hospital between 2003 and 2011 was ∼150 per 100,000 people in the United States [4]. The causes of PAD are multiple, related to general (wound, contusion, compression) or local arterial injuries (atherosclerosis, embolic events, thrombosis, dissections of the arterial wall) and result finally in vessel obstruction leading to limb ischemia [5]. Lower limb ischemia requires surgical revascularization, possibly associated with thrombolysis, but revascularization might be impossible in some cases, highlighting the need for new therapeutic approaches [2,6].

Recent improvement in the knowledge of PAD pathophysiology might be useful to open new therapeutic approaches. Thus, skeletal muscle mitochondrial dysfunctions, together with increased reactive oxygen species (ROS) production are key factors. Mitochondria play a central role in cell homeostasis, since they are the primary sites of energy production through ATP synthesis. Besides cellular energy metabolism adaptation, such organelles also modulate the production of ROS and consequently cell apoptosis, which is increased when mitochondrial calcic retention capacity (CRC) is impaired [7,8,9,10]. Interestingly, if ischemia per se is clearly deleterious and urges to perform revascularization procedures, the reperfusion period is also responsible for deleterious effects relying importantly on the amount of ROS released. Thus, after aortic cross-clamping, increased oxidative stress precedes mitochondrial dysfunction [11].

Ischemic preconditioning generally demonstrated to be protective in lower limb ischemia-reperfusion [12,13]. Ischemic post-conditioning can also reduce oxidative stress and preserve antioxidant defense in an experimental model of aortic clamping [14] but it has also been shown to be deleterious [15]. These data pave the way to develop pharmacological approaches. Cyclosporine A demonstrated promising results by protecting muscle in young rats but was disappointing when investigating old animals submitted to lower limb ischemia-reperfusion (IR) with tourniquet use [16,17]. Interestingly, confirming data previously obtained in the setting of myocardial infarction [18], we observed that the cardiac hormone brain natriuretic peptide (BNP) reduced skeletal muscle mitochondrial dysfunction and oxidative stress after acute lower limb IR [19]. Since BNP main actions are mediated by an increase in the second intracellular messenger cyclic guanylyl monophosphate (cGMP), another approach aiming to reduce cGMP degradation might be interesting.

In this view, phosphodiesterase inhibition deserves to be investigated. Cyclic nucleotide phosphodiesterase families (PDEs) control cyclic nucleotide levels and play a major role in the control of normal and pathological cellular signaling [20,21,22]. Particularly, the PDE5 family hydrolyzes specifically cGMP, and its inhibition increases cGMP levels. Sildenafil, a well-known PDE5 inhibitor, is widely used in humans mainly for treating pulmonary hypertension and erectile dysfunctions [23]. It also induced protective effects during ischemia-reperfusion on several organs [24,25,26,27,28,29], supporting its potential usefulness in IR settings. PDE5 has been also shown to be expressed in skeletal muscle [21,30], and, accordingly, sildenafil reduced muscle oxidative stress and/or increased muscle angiogenesis and reduced inflammation 7 or 30 days after either femoral artery removal or unilateral hindlimb IR [31,32,33].

The aim of this study was, therefore, to investigate whether pharmacological pre-conditioning with the PDE5 inhibitor might, through its anti-oxidant effect, protect skeletal muscle mitochondrial oxidative capacity and calcium retention capacity, using the model of tourniquet-induced lower limb IR.

## 2. Material and Methods

### 2.1. Animals

Eighteen male Swiss mice (12–16 week old), provided by JANVIER Labs (Saint Berthevin, France), were housed in a thermo-neutral environment at 22 ± 2 °C on a 12 h day/night cycle and were provided food and water ad libitum. The protocol was approved by the Regional Committee of Ethics in Animal Experimentation of Strasbourg (C.R.E.M.E.A.S) and the Ministry of Higher Education and Research (CREMEAS n°2018022716192465v3), and the animal care complied with the Guide for the Care and Use of Laboratory Animals, Institute of Laboratory Animal Resources, Commission on Life Sciences, National Research Council. Washington: National Academy Press, 1996.

### 2.2. Experimental Design

**Figure 1:**
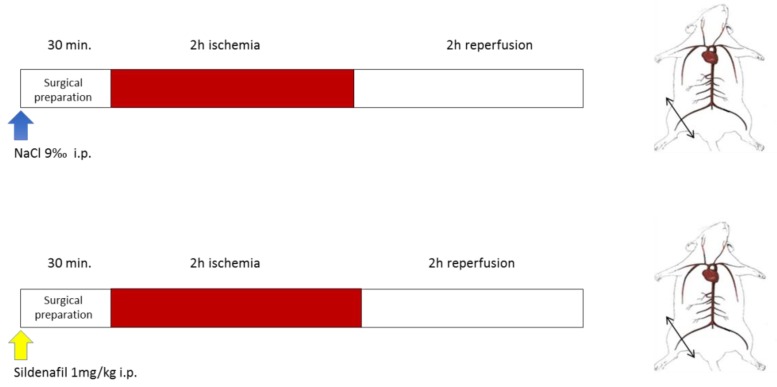
Experimental design. Top (NaCl): Ischemia-reperfusion (IR) animals underwent 2 h of unilateral hind limb tourniquet ischemia (red bar), followed by 2 h of reperfusion (open bar). The right, non-ischemic hind limb served as a control, and Nacl was administered ip 30 min before ischemia. Bottom (Sildenafil + IR): the same protocol was performed, but 1mg/kg sildenafil/NaCl ip was administered.

All mice were subjected to 2 h ischemia through a tourniquet placed on the right hindlimb, followed by 2 h reperfusion (Figure 1). The left non-ischemic hindlimb served as a control, since previous data demonstrated that unilateral limb ischemia did not significantly affect the contralateral limb [34]. Thirty minutes before ischemia, sham mice (n = 8) received intraperitoneal NaCl 9‰ (5 µL/g), and sildenafil mice (n = 10 mice) received intraperitoneally sildenafil/NaCl 9‰ (1 mg/kg). Mice were then placed in a hermetic anaesthetic induction cage, ventilated with a gas mixture of 4% isoflurane (AERRANE^®^, BAXTER S.A.S.) and oxygen and placed on heating blankets (MINERVE^®^, Esternay, France) at 37 °C. Spontaneous ventilation was allowed through an oxygen-delivering mask, with different concentrations of isoflurane depending on the surgical stage (2% during painful stimuli and 1% during latent periods). At the end of the experiment, left and right gastrocnemius muscles were dissected and immediately immersed in Krebs solution at 4 °C for the extemporaneous analyses of mitochondrial functions.

### 2.3. Mitochondrial Respiratory Chain Complex Activities

Muscle oxygen consumption was determined using a high-resolution oxygraph (Oxygraph 2K, Oroboros instruments, Innsbruck, Austria), in 2 ml of Miro5 + Cr thermostated at 37 °C, containing EGTA (0.5 mM), MgCl_2_ (3 mM), K lactobionate (60 mM), taurine (20 mM), KH_2_PO_4_ (10 mM), HEPES (20 mM), sucrose (110 mM), creatine (20 mM), BSA (1 g/L).

Permeabilized fibers were placed in chambers to record the basal oxygen consumption (V_0_) with glutamate (10 mM) and malate (2.5 mM). Then, maximal respiration rate (V_Max_) was measured under continuous stirring in the presence of saturating amount of ADP (2 mM) as a phosphate acceptor. Succinate injection (25 mM, V_succ_) allowed the activation of all complexes (I, II, III, IV, V). Finally, ascorbate (0.5 mM) and N, N, N ‘, N’-tetramethyl-p-phenylenediamine dihydrochloride (TMPD, 0.5 mM) were injected, V_asc/TMPD_ representing the complex IV contribution. Results were expressed as pmol/sec/mg wet weight.

### 2.4. Calcium Retention Capacity (CRC) Measurements in Gastrocnemius Ghost Fibers

The time to the opening of the mitochondrial permeability transition pore (mPTP) following Ca^2+^ challenge (5 µL of Ca^2+^, 1 mM, pulses performed every 5 min) was determined in permeabilized “ghost” muscle fibers [23]. The amount of Ca^2+^ needed to trigger a massive Ca^2+^ release by the mitochondria due to mPTP opening was calculated from a standard curve relating [Ca^2+^] to the fluorescence of calcium green and expressed as μmol/mg dry weight.

### 2.5. Production of Reactive Oxygen Species Using Electron Paramagnetic Resonance

Immediately after harvesting, gastrocnemius muscles were placed in Krebs solution containing NaCl 99 mM, KCl 4.69 mM, CaCl_2_ 2.5 mM, MgSO_4_ 1.2 mM, NaHCO_3_ 25 mM, KH_2_PO_4_ 1.03 mM, D(+) glucose 5.6 mM, Na-Hepes 20 mM, deferoxamine 25 µM, and DETC 5 µM. Tissues were cut into 1 to 2 mm^3^ slices and incubated for 30 min with 1-hydroxy-3-methoxycarbonyl-2, 2, 5, 5-tetramethylpyrrolidine HCl (CMH) in a thermo-regulated incubator (37 °C) under controlled pressure (20 mmHg) and gas mix (N_2_: 97.8%, O_2_: 2.8%) (Noxygen^®^, Germany). EPR spectroscopy (Bruker Win-EPR^®^, Bruker Analytik, GmbH) was used to determine ROS production, expressed in µmol/min/mg dry weight.

## 3. Statistical Analysis

All results were expressed as means ± SEM. Data were analysed using Prism software (GraphPad Prism 5, Graph Pad Software, Inc., San Diego, CA, USA), and differences between groups were assessed using two-way ANOVA test, followed by the Bonferroni post-test.

To determine the precise *p* value when considering ROS, paired t–test was performed. A *p* value < 0.05 was considered significant.

## 4. Results

### 4.1. Lower-Limb Ischemia-Reperfusion Impaired Skeletal Muscle Mitochondrial Respiration and Calcium Retention Capacity and Tended to Increase ROS Production

#### 4.1.1. Mitochondrial Respiration

**Figure 2:**
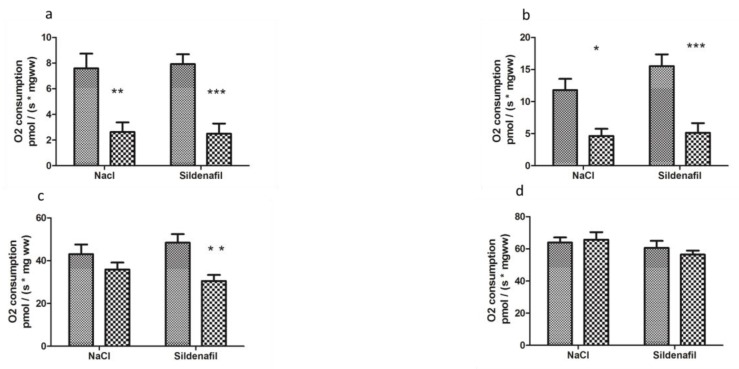
Mitochondrial respiration in ischemic and non-ischemic gastrocnemius in mice treated (n = 10) or not (n = 8) with sildenafil; (**a**): V_0_ corresponds to the basal O_2_ consumption, with glutamate and malate as substrates. (**b**): V _max_ corresponds to the ADP-stimulated respiration, with glutamate and malate as substrates. (**c**): V_succ_ represents the activation of all complexes (I, II, III, IV, V). (**d**): V_asc/TMPD_ represents the complex IV contribution to the global mitochondrial respiratory rate. For both NaCl and Sildenafil groups: left column, non-ischemic contralateral limb. Right column: Ischemic limb. Results are expressed as means ± SEM. *: *p* < 0.05; **: *p* < 0.01 and ***: *p* < 0.001, as compared to the contralateral limb of the same group.

Two h ischemia followed by 2 h reperfusion significantly decreased V_0_ (basal oxygen consumption rate) of ischemic limbs, as compared to contralateral non-ischemic limbs (7.58 ± 1.16 vs 2.63 ± 0.74 pmolO_2_/s/mg wet weight (ww), −62.8 ± 9.5%, *p* < 0.01, in non-ischemic and ischemic limbs, respectively, Figure 2).

Maximal mitochondrial respiration rate, V_max_, which corresponds to complexes I, III, IV, and V of the mitochondrial respiratory chain, decreased from 11.79 ± 1.74 to 4.65 ± 1.11 pmolO_2_/s/mg ww (−50.2 ± 16.3% *p* < 0.05, Figure 2).

V_succ_, (activity of complexes I, II, III, IV, and V) decreased from 43.03 ± 4.55 to 35.85 ± 3.24 pmolO_2_/s/mg ww (−11.6 ± 9.9%, Figure 2).

Finally, V_asc/TMPD_, reflecting more specifically the activity of complex IV, was not significantly modified (63.89 ± 3.19 vs 65.68 ± 4.70 pmolO_2_/s/mg ww, Figure 2.

#### 4.1.2. Calcium Retention Capacity (CRC)

**Figure 3:**
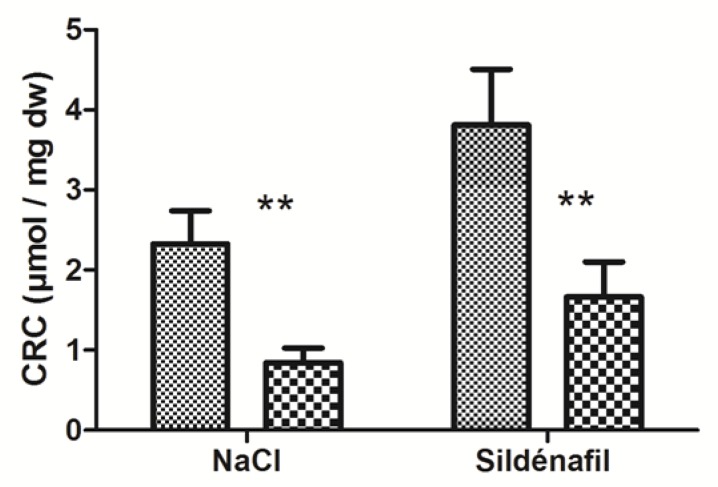
Calcium retention capacity (CRC) in ischemic and non-ischemic gastrocnemius in mice treated (n = 7) or not (n = 9) with sildenafil. For both NaCl and Sildenafil groups: left column, non-ischemic contralateral limb. Right column: Ischemic limb. Results are expressed as means ± SEM. **: *p* < 0.01.

IR significantly decreased skeletal muscles’ CRC in ischemic limbs (2.33 ± 0.41 vs 0.84 ± 0.18 μmol/mg dry weight (dw); −61.1 ±6.8 % *p* < 0.01, Figure 3) in ischemic vs non-ischemic limbs.

#### 4.1.3. Oxidative Stress

**Figure 4:**
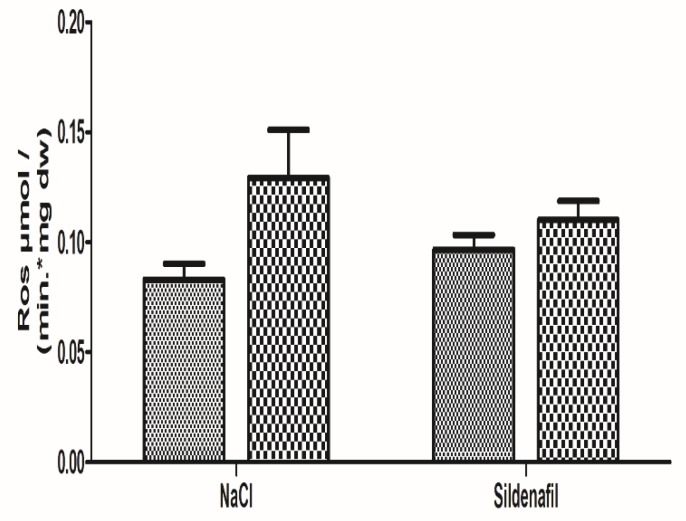
Reactive Oxygen species (ROS) production in ischemic and non-ischemic gastrocnemius in mice treated (n = 8) or not (n = 8) with sildenafil. ROS level was obtained using electron paramagnetic resonance. For both NaCl and Sildenafil groups: left column, non-ischemic contralateral limb. Right column: Ischemic limb. Results are expressed as means ± SEM. *p* = 0.08 in the NaCl group.

Production of ROS was 0.08 ± 0.01 in the non-ischemic limbs and 0.13 ± 0.02 μmol/min/mg dw in the ischemic limbs (+64.3 ± 31.9%; *p* = 0.08), as seen in Figure 4.

### 4.2. Sildenafil Did Not Protect Skeletal Muscle Mitochondrial Respiration and Calcium Retention Capacity, Albeit Tending to Reduce ROS Production

#### 4.2.1. Mitochondrial Respiration

In sildenafil-treated mice, V_0_ was reduced (7.70 ± 0.64 vs 2.83 ± 0.80 pmolO_2_/s/mg ww; −62.9 ± 10.8% *p* < 0.001, Figure 2) in ischemic limbs.

V_max_ decreased from 15.29 ± 1.75 to 5.74 ± 1.47 pmolO_2_/s/mg ww, (−63.3 ± 9.2% *p* < 0.001, Figure 2).

V_succ_ decreased from 48.37 ± 4.02 to 31.63 ± 2.65 pmolO_2_/s/mg ww in ischemic limbs (−30.4 ± 7.9% *p* < 0.01, Figure 2).

V_asc /TMPD_ was 61.49 ± 3.94 in contralateral limbs and 56.72 ± 2.43 pmolO_2_/s/mg ww in ischemic limbs, *p* was not significant (Figure 2).

#### 4.2.2. Calcium Retention Capacity (CRC)

In sildenafil-treated mice, CRC decreased from 3.81 ± 0.70 (non-ischemic limbs) to 1.66 ± 0.44 μmol/mg dw (ischemic limbs), (−55.2 ± 7.6%, with *p* < 0.01, Figure 3).

#### 4.2.3. Oxidative Stress

Production of ROS in sildenafil-treated mice was 0.10 ± 0.01 in the non-ischemic limbs and 0.11 ± 0.01 μmol/min/mgdw in the ischemic limbs (+21.9 ± 16.6%, *p* = 0.34), as shown in Figure 4.

The sildenafil-related reduction in ROS production (−42.4%, as compared to the NaCl group) failed to reach statistical significance.

## 5. Discussion

The main findings of this study are that lower limb IR impaired mitochondrial oxidative capacity and decreased calcium retention capacity in skeletal muscle. Further, albeit reducing ROS production, acute preconditioning with the phosphodiesterase 5 inhibitor sildenafil did not reduce IR-induced muscle injuries.

### 5.1. Effects of Ischemia-Reperfusion

Using different substrates, we observed that the functional activities of the main mitochondrial respiratory chain complexes were impaired by IR. The gastrocnemius muscle was chosen in view of its susceptibility to IR injuries [35,36], allowing it to be a key target when investigating new therapeutic approaches. Thus, main mitochondrial complexes showed a significant decrease in their oxidative capacity, and ROS production tended to be increased. These data are in accordance with results previously obtained with lower limb IR models, secondary to aortic ligation or unilateral leg IR, which demonstrated reduced skeletal muscle oxidative capacities together with increased ROS production [13,14,15,16,17,19,37,38]. Accordingly, oxidative damage in the gastrocnemius has been observed in patients with peripheral artery disease [39]. Interestingly, IR also induced a reduction in mitochondrial calcium retention capacity. This corresponded to a rapid opening of the mitochondrial permeability transition pore, leading thereby to cell apoptosis.

### 5.2. Effects of Sildenafil

Several studies previously reported a protective effect of sildenafil in IR settings. Thus, sildenafil protection was reported during acute cardiac IR in mouse [40], in the human heart [25], as well in the kidney [26], lung [27], liver [28], and brain [29]. Further, another phosphodiesterase 5 inhibitor, vardenafil, improved vascular graft function [41]. Taken together and considering the fact that PDE5 expression has been observed in skeletal muscles [21,30,42], these data suggest that PDE5 might also be implicated in skeletal muscle IR.

Accordingly, chronic administration of sildenafil improved ischemia-induced neovascularization in hypercholesterolemic apolipoprotein E-deficient mice, reduced TNF alpha staining in the femoral artery, and reduced inflammation and oxidative stress in skeletal muscle [31,32,33].

Nevertheless, our study shows that, although tending to decrease ROS production in line with literature data obtained both in skeletal and in cardiac muscles [31,43], sildenafil did not overcome the deleterious effects of acute IR on skeletal muscle. This was unexpected, since we previously observed that BNP, also known to increase the second messenger cGMP, protected skeletal muscle in a similar setting [19]. 

One explanation might be that chronic treatment might be mandatory. Indeed, beneficial effects of sildenafil were observed after daily, weekly, or even longer administration [31,32,33]. However, acute sildenafil administration also demonstrated some degrees of protection [44,45,46]. In these cases, the PDE5 inhibitor was administered either prior to ischemia or 30 min before reperfusion and after release of the clamps [44,45,46]. Similarly, we administrated sildenafil 30 minutes before ischemia to allow a large diffusion in muscles. Indeed, maximal concentration occurred 1 h after a single intravenous administration, and the half-life was around 0.4–1.3 h in rodents [47]. These studies, nevertheless, investigated mainly environmental changes in muscles, focusing on the inflammatory status. Thus, muscular changes consisted principally in reduced inflammatory cells and TNF alpha staining [44,45]. Necrosis was not reduced when using sildenafil alone [45]. Particularly, no previous data have been reported concerning the specific effect of sildenafil on IR-induced skeletal muscle mitochondrial dysfunctions, but, interestingly, sildenafil reduced caspase 3, which is considered a marker of apoptosis, only at a late stage (24 h) of reperfusion. No significant change was observed at 4 h of reperfusion [46].

In line with our results, Nio et al. [48] reported only a slight mRNA expression of PDE5 in gastrocnemius and, contrary to data obtained in the lung and the heart, oral sildenafil at a single dose of 30 mg/kg did not increase cGMP in such muscle [48]. Thus, a low physiological level of intracellular cGMP and/or a low amount of PDE type 5 in the gastrocnemius muscle might suggest a longer or greater exposure to sildenafil in order to protect skeletal muscle.

Thus, higher sildenafil doses might deserve further discussion. Higher dosages (40 and 10 mg/Kg) of oral sildenafil have been previously used [31,33]. However, 1 mg/Kg sildenafil also showed beneficial effects using different administration modalities (per os, ip, iv) [32,44,45,46]. In our setting, administering 1 mg/Kg sildenafil for two times and combining pre- and post-conditioning might deserve further investigations.

## 6. Conclusions

Lower limb IR significantly impairs skeletal muscle mitochondrial functions. However, although beneficial in other IR settings and despite its tendency to decrease ROS production, acute pharmacologic preconditioning with the phosphodiesterase 5 inhibitor sildenafil did not reduce IR-induced impairments in mitochondrial oxidative and calcium retention capacities in skeletal muscle. Further studies will be useful to further determine the functional roles of PDE and cGMP and to investigate mitochondria-targeted antioxidants in normal and ischemic skeletal muscles [49].
